# Composite Membranes of Recombinant Silkworm Antimicrobial Peptide and Poly (L-lactic Acid) (PLLA) for biomedical application

**DOI:** 10.1038/srep31149

**Published:** 2016-08-09

**Authors:** Zhi Li, Xuan Liu, Yi Li, Xiqian Lan, Polly Hangmei Leung, Jiashen Li, Gang Li, Maobin Xie, Yanxia Han, Xiaofen Lin

**Affiliations:** 1 Chongqing Engineering Research Center of Biomaterial Fiber and Modern Textile, College of Textiles and Garments, Southwest University, Chongqing, China; 2 Institute of Textiles and Clothing, The Hong Kong Polytechnic University, Hung Hom, Kowloon, Hong Kong; 3 School of Materials, The University of Manchester Manchester M13 9PL, UK; 4 State Key Laboratory of Silkworm Genome Biology, Southwest University, Chongqing, China; 5 Department of Health Technology and Informatics, The Hong Kong Polytechnic University, Hung Hom, Kowloon, Hong Kong; 6 National Engineering Laboratory for Modern Silk, College of Textile and Clothing Engineering, Soochow University, Suzhou, China

## Abstract

Antimicrobial peptides, produced by innate immune system of hosts in response to invading pathogens, are capable of fighting against a spectrum of bacteria, viruses, fungi, parasites and cancer cells. Here, a recombinant silkworm AMP Bmattacin2 from heterologous expression is studied, indicating a broad spectrum of antibacterial activity and showing selective killing ability towards skin and colon cancer cells over their normal cell counterparts. For the purpose of biomedical application, the electrospinning fabrication technique is employed to load Bmattacin2 into PLLA nanofibrous membrane. In addition to a good compatibility with the normal cells, Bmattacin2 loaded nanofibrous membranes demonstrate instant antibacterial effects and sustained anticancer effects. The cancer cell and bacteria targeting dynamics of recombinant Bmattacin2 are investigated. With these characteristics, PLLA/Bmattacin2 composite membranes have a great potential for developing novel biomedical applications such as cancer therapies and wound healing treatments.

Antimicrobial peptides (AMPs) are a group of peptides with the ability of killing the microbial invaders, which is a significant way of the innate immune systems in most organisms^1^. These peptides are generally less than 150–200 amino acid residues. Despite the diversity of size, and primary and secondary structures, AMPs share common characteristics in that almost all peptides possess cationic surface charges and a significant proportion of hydrophobic residues to form a membrane-bound amphipathic conformation^2^. The former characteristic is obviously responsible for the reason why there is electrostatic attraction between AMPs and target cells. The latter one plays a key role in the mechanism of AMPs’ action after the initial attraction between AMPs and cell membrane. In addition, AMPs generally possess a series of common features such as thermal stability and broad-spectrum antimicrobial activity. Some of AMPs have been demonstrated to inhibit the replication of some kinds of viruses including human immunodeficiency virus (HIV)^3^,^4^ and influenza A virus^5^. The activity of anticancer cell and antiparasite of AMPs is also reported^6^,^7^. In this study, considering the differential membrane properties, especially the electrostatic characters of microbial and human cells^8^,^9^, two categories of bacteria and two pairs of human cells (normal and cancerous) from skin and colon tissue were evaluated for their susceptibility to Bmattacin2. Measurements of zeta potential of cells were adopted to monitor the change of surface charge of cells^10^,^11^,^12^.

In silkworm, there are seven kinds of AMPs, including attacin, cecropin, defensin, enbocin, lebocin, moricin and gloverin. In terms of attacin, two proteins named attacin1 and attacin2 were identified and their cDNA sequences have been revealed^13^,^14^. Traditional method for obtaining the silkworm AMPs is to isolate them directly from fat body or hemolymph of organism; however, it is difficult and time-consuming. Gene clone and expression in heterologous system is a process of protein engineering technology, which was verified as an effective way to derive large scale of useful and functional proteins.

Functional materials with anti-oxidation, anti-inflammatory, antimicrobial and anticancer activity were used to prepare the tissue engineered scaffolds together with natural and synthetic materials; however, the toxic effect to normal cells at their efficient concentration was observed. Mohiti-Asli, M. *et al.* used silver microparticles (AgMPs) as an alternative to silver nanopaticles (AgNPs) and loaded them to PLA nanofibers for control release. Although it exhibited inhibitive effect on *Staphylococcus aureus* (*S. aureus*), the cytotoxicity to human epidermal keratinocyte could not be avoided and it was still obvious^15^. Similar study by Jin, G. *et al.* indicated that poly(L-lactic acid)-co-poly(ϵ-caprolactone) nanofibres loaded a concentration of 0.25% silver nanoparticles could fight against *S. aureus* and *Salmonella enterica*. Moreover, increasing concentration of silver nanoparticles resulted in the enhanced antibacterial activity of scaffold; however, the cytotoxicity to human skin fibroblast increased simultaneously^16^. Some drugs, such as 5-fluorouracil (5-FU), are also loaded to the scaffolds for drug release and cancer therapy^17^; however, their harm to normal cells cannot be ignored. Thus, to solve this problem and develop a novel scaffold with higher antibacterial and anticancer function as well as lower cytotoxicity for biomedical application, the recombinant silkworm Bmattacin2, as a bioactive material, was loaded to the nanofibrous scaffolds by electrospinning. The investigation indicated these electrospun PLLA/Bmattacin2 nanofibrous membranes have a great potential for biomedical application such as cancer therapy, wound dressing and healing.

## Results

### Cancer cell targeting ability of Bmattacin2

**Figure 1:**
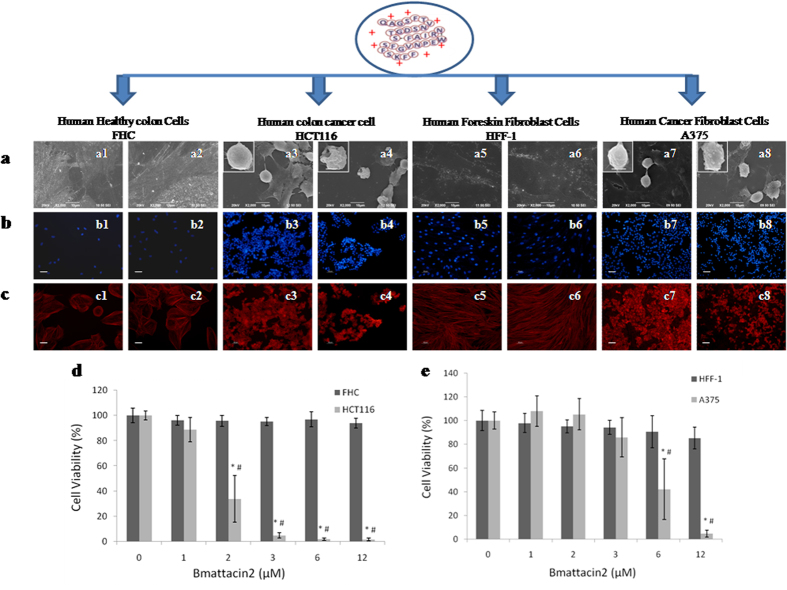
Cancer cell-targeting ability of Bmattacin2. Cells were treated with culture medium (odd numbers) or 2 μM of Bmattacin2 (even numbers) for 24 hours. (**a**) Cell surface morphology was observed by scanning electronic microscopy. Bar = 10 μm. (**b**) The cellular nucleus was stained by DAPI. Bar = 50 μm. (**c**) The cellular skeleton (actin) was stained by TRITC-phalloidin. Bar = 50 μm. (**d**) Human colon normal cells (FHC) and cancer cells (HCT116) were subjected by Bmattacin2 from 1 to 12 μM for 24 hours and cell viabilities were evaluated by MTS assay. (**e**) Fetal foreskin fibroblasts (HFF-1) and skin malignant melanoma cells (A375) served as another pair of comparison.

By using a heterologous system *E. coli* BL21, an AMP gene from silkworm *Bombyx mori*, *Bmattacin2*, was expressed. The recombinant Bmattacin2 has a molecular size of 22 KD (Supplementary Fig. S1). Further, it was purified (Supplementary Fig. S1). We evaluated the effects of Bmattacin2 on the viability of two cancerous cells (malignant melanoma cells, A375 and human colorectal cancer cells, HCT116) and together with their normal counterparts (human foreskin fibroblasts, HFF-1 and fetal human colon cells, FHC). Compared with that of the unaffected normal cells (Fig. 1a2), the surface morphology of two cancer cells (Fig. 1a4) was severely disrupted in the presence of 2 μM of Bmattacin2 after 24 hours of incubation. Nuclear fragmentation or chromatin condensation (Fig. 1b4) and the disconnected cytoskeleton of A375 and HCT116 (Fig. 1c4) were apparent compared with the normal cells. The IC_50_ of Bmattacin2 towards A375 and HCT116 were 5.23 and 1.70 μM respectively, in the low micromolar range. The viability evaluation (Fig. 1d,e) further supported the potential for Bmattacin2 that selectively kills A375 and HCT116 cancer cells while sparing their normal counterpart under 12 μM.

### Antibacterial effects of Bmattacin2

**Figure 2:**
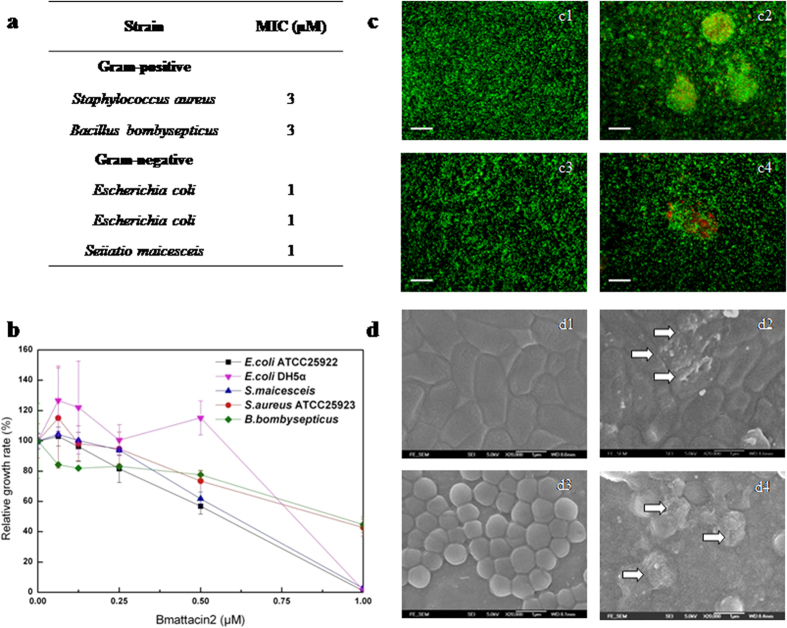
Antibacterial effects of Bmattacin2. (**a**) Antimicrobial activities (MICs) of Bmattacin2 against a panel of Gram-positive and Gram-negative bacteria (*E. coli* DH5α, *E. coli* ATCC25922, *S. maicesceis*), including three Gram-negative bacteria and two Gram-positive bacteria (*S. aureus* ATCC25923 and *B. bombysepticus*). (**b**) Bmattacin2-mediated growth inhibition of bacteria was conducted by recording the OD value at 600 nm after incubation of 18 hours. The growth rates among the different bacteria strains in a range of concentration of Bmattacin2 were normalized by control group (**b**). Data was expressed as mean with standard deviation of at least three replicates. (**c**) Live/dead staining of *E. coli* ATCC25922 and *S. aureus* ATCC25923 (green refer to live cells, red refer to dead cells) after treated with Bmattacin2 at their MIC for 18 hours respectively. Bar = 50 μm. (**d**) The effects of Bmattacin2 against surface of *E. coli* ATCC25922 and *S. aureus* ATCC25923 were evaluated through SEM observation. Bar = 1 μm.

The expression of Bmattacin2 in silkworm is induced by foreign invaders. Two kinds of bacteria which are usually found in infected silkworms, *S. maicesceis* and *B. bombysepticus*, together with together with three common bacterial strains in the lab, *E. coli* ATCC25922, *E. coli* DH5α and *S. aureus* ATCC25923, were used to test the antibacterial activity of recombinant Bmattacin2. MIC assay indicated that Bmattacin2 was active against Gram-negative bacteria (*E. coli* DH5α, *E. coli* ATCC 25922, and *S. maicesceis*) and Gram-positive bacteria (*S. aureus* ATCC 25923 and *B. bombysepticu*) (Fig. 2a). The proliferation of these Gram-negative and Gram-positive bacteria stains were all inhibited by Bmattacin2 in a dose dependent manner (Fig. 2b). As shown in Fig. 2c, a fluorescence live/dead stain indicated that Bmattacin2 killed *E. coli* and *S. aureus* bacteria at the corresponding MICs. Multiple holes of various shapes on the surface of cell walls of *E. coli* were seen after incubation with Bmattacin2. The adverse effect on *S. aureus* ATCC25923 caused by Bmattacin2 is the loss of cellular membrane and cell debris (Fig. 2d). A broad antibacterial spectrum of the recombinant Bmattacin2 is clearly observed.

### Targeting Dynamics

**Figure 3:**
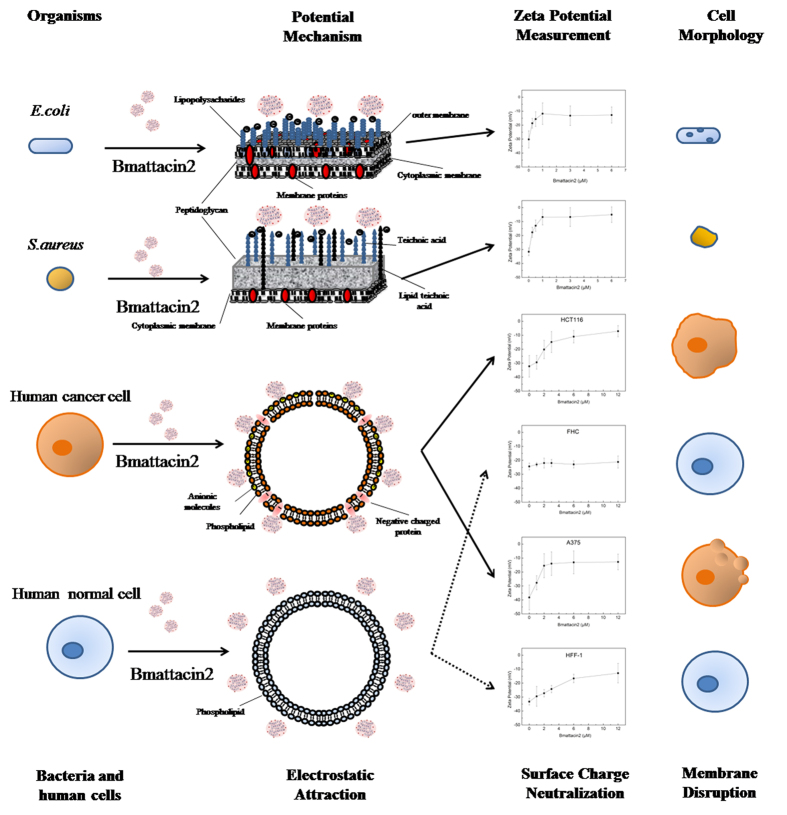
Potential mechanisms of antibacterial activity and cancer cell-targeting effects of Bmattacin2. Bmattacin2 was evaluated as a cationic protein at physiological pH value with amphipathic conformation. Antibacterial activity of Bmattacin2 may due to the electrostatic attraction. Cell surface charge was assessed by monitoring zeta potential of cells. Zeta potential of *E. coli*, *S. aureus*, FHC, HCT116, HFF-1, A375 were measured after cells were incubated in the absence or presence of Bmattacin2 for 30 minutes. The corresponding morphology changes of cells were described.

One of the most commonly recognized attack mechanisms of AMPs is membrane disruption when these peptides encounter bacteria and cells. To improve our understanding of the interaction between Bmattacin2 and different cells, we characterized the surface charge of these bacteria and human cells by measuring zeta potential value in the absence and presence of Bmattacin2 (Fig. 3). It is believed that the positive turning of zeta potential reflects an electrostatic interaction of the negatively charged cell surface with positively charged peptides^10^,^11^,^12^. In terms of bacteria, the electrostatic interaction is the primary step of proposed hypotheses of AMP’s working mechanisms. Barrel stave model, carpet model or toroidal pore model are proposed to relate to the death of bacteria. For cells, the increasing Bmattacin2 caused a rise of zeta potential of all tested cells except for FHC, indicating a weak electrostatic interaction between Bmattacin2 and this normal fetal colon cell. The surface charge neutralization of HFF-1 cells was also observed, though no further adverse effects were found afterwards. It is possible that the binding effect did not reach a saturation status to initiate a lethal effect for HFF-1 cells. Another possibility is that the electrostatic attraction contributed to the effect of Bmattacin2 interact with the encountered cells^8^,^9^,^18^,^19^, further engagement of protein and membrane is also required to trigger the lethal effects^10^,^11^. FITC-labeled Bmattacin2 showed preferential binding affinity to cancerous cells (Supplementary Fig. S2), demonstrating a stabilized involvement between Bmattacin2 and the membrane of cancerous cells^20^,^21^ reinforced the possibility of Bmattacin2 in selective killing of cancerous cells.

There are two kinds of attacins in silkworm. Both attacins are the glycine-rich AMPs and their domains with these glycine residues are weakly-conserved during evolution just like the attacin family from other species^22^. The characterization showed Bmattacin2 has as high as 25% of α-helix in its secondary structure, which is verified as a structure likely correlated with the antimicrobial activity^23^. Meanwhile, Bmattacin2 has a net charge of 4.1 at pH 7.0 which is possibly due to the accumulation of cationic amino acids. The possession of the positive charge in the functional structure of Bmattacin2 is likely a reason for electrostatic interaction. For bacteria, the great amount of lipopolysaccharides (LPS) or teichoic acid (TA) presented on the cell wall brought negative surface charge to microbial outer membrane, and for cancerous cells, high expression of phosphatidylserine, glycolipid and glycoprotein or chaperone proteins also contributed to the negative charge density^24^,^25^,^26^,^27^,^28^. For our investigation, these properties are believed lead to an electrostatic interaction between cationic Bmattacin2 and negative charged membrane of bacterial or cancerous cells, which is a necessary but not exclusive prerequisite for the selective killing of this recombinant AMP.

### Composite membranes with PLLA and Bmattacin2

**Figure 4:**
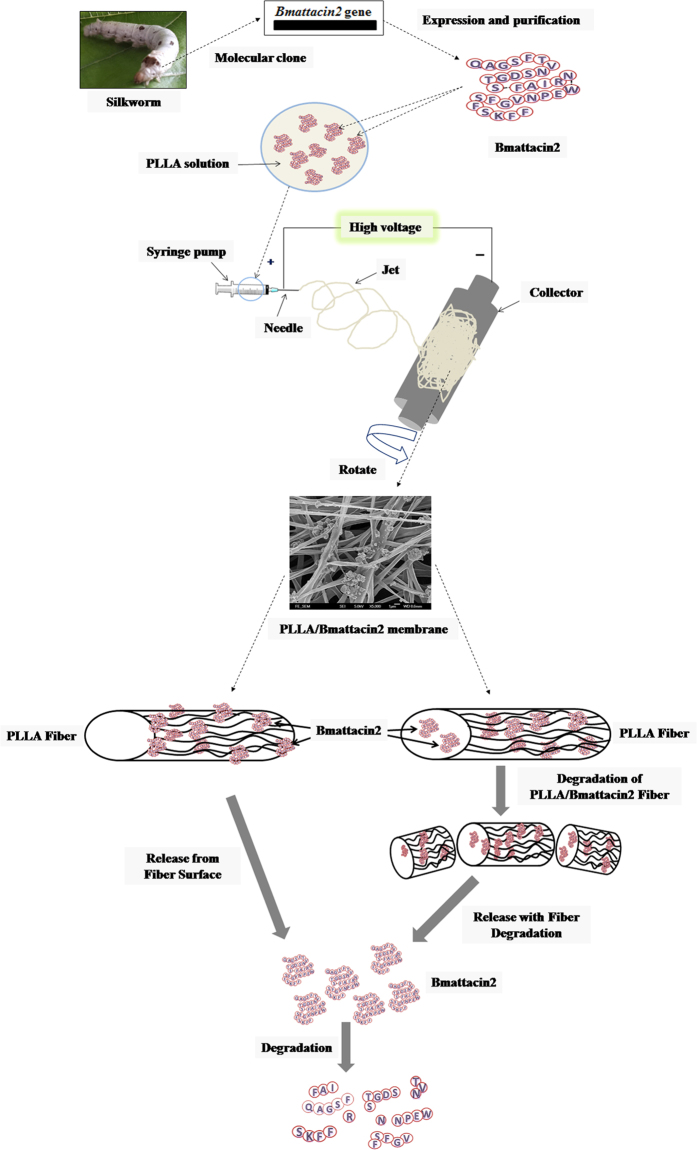
The load of Bmattacin2 on the PLLA membrane and the release of Bmattacin2 from the PLLA/Bmattacin2 membrane.

Multiple strategies have been applied to integrate AMPs with suitable substrates to overcome the drawbacks comes from their vulnerable stability in solution and the adverse effects towards host cells at their efficient concentration^29^,^30^,^31^,^32^,^33^. Using electrospinning to introduce functional biological molecules into nanofibrous scaffolds on the surface or interior of the fibers, the long-term release of protein or peptides could be preserved (Fig. 4)^34^,^35^,^36^,^37^. In this study, PLLA membrane and PLLA membrane loaded with the concentration of Bmattacin2 2% were prepared by electrospinning. In order to evaluate microstructure of these electrospun composite membranes, FTIR spectrum was used. Three peaks representing amide I, amide II and amide Ш at 1634 cm^−1^, 1531 cm^−1^, and 1258 cm^−1^ respectively were detected for samples of Bmattacin2 and the first peak at 1634 cm^−1^ was also evident in the spectra of PLLA/Bmatttacin2 nanomembrane although the latter two peaks were too weak to be observed. In addition, the peak at 1757 cm^−1^ is a characteristic peak of PLLA which was also found in the spectra of PLLA/Bmatttacin2 nanomembrane (Supplementary Fig. S3), which confirmed that the electrospun PLLA/Bmatttacin2 nanomembrane consisted of PLLA and Bmattacin2. The electrospun PLLA/Bmattacin2 membrane had a wide range of diameters from 170 nm to 2180 nm (Supplementary Fig. S4). With these diameters, PLLA/Bmattacin2 had a smaller tensile strength of around 2.7 ± 0.3 MPa; however, the tensile modulus of PLLA/Bmattacin2 was 45.7 ± 13.2 MPa and the tensile strain was 24.2 ± 3.9% which indicated this electrospun PLLA/Bmattacin2 membrane was fit for use as a scaffold in human skin TERM (Supplementary Table S1). Further, the investigation of the release property of Bmattacin2 from the membrane (Supplementary Fig. S5) revealed that although most of the Bmattacin2 rapidly dissolved after the membrane was immersed and the low concentration of Bmattacin2 could be retained after 48 hours.

### Characterization of PLLA/Bmattacin2 membranes

**Figure 5:**
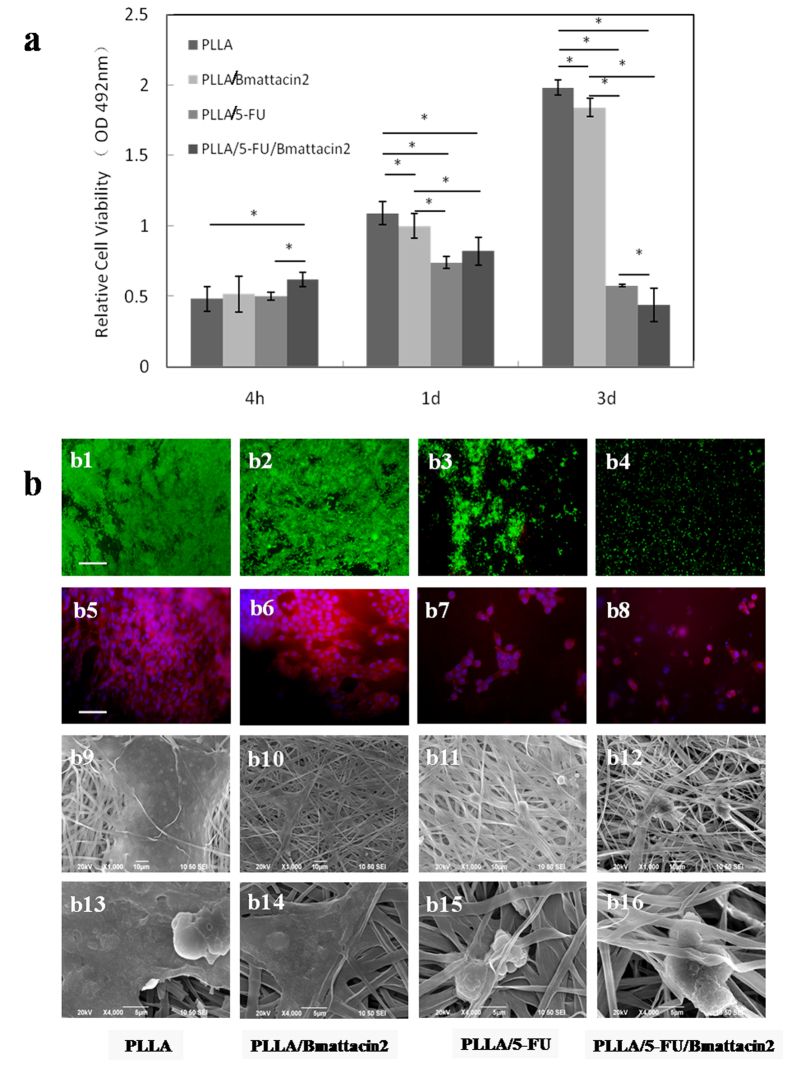
Anticancer activity of electrospun composite membranes. (**a**) Cell viability of HCT116 after seeding on fabricated composite membranes for 4 hours, 1 day and 3 days. Data is presented as the mean with standard deviation from triplicate determinations, *p < 0.05. (**b**) Observation of HCT116 cells cultured on the surface of fabricated composite membranes for 3 days. (b1–b4) Live/dead cell staining. Bar = 50 μm. (b5–b8) Fluorescent staining of cells by DAPI/FITC. Bar = 50 μm. (b9–b16) SEM observation.

To develop a novel scaffold with antibacterial and anticancer effect for biomedical application, PLLA/Bmattacin2, PLLA/5-FU, and PLLA/5-FU/Bmattacin2 membranes were prepared by electrospinning. As shown in Fig. 5a, viability of HCT116 on PLLA/Bmattacin2, PLLA/5-FU and PLLA/5-FU/Bmattacin2 were gradually suppressed at 1 day. The additionally lower OD_492_ value of cells on PLLA/5-FU/Bmattacin2 at 3 days suggested that Bmattacin2 plays a synergetic role in the anticancer cell effect of fabricated scaffolds. The visualization of cell status on 3 days indicated a similar trend that PLLA/5-FU/Bmattacin2 was found to obtain the least living cells (Fig. 5b4), affected integrity of cytoskeleton and nucleus (Fig. 5b8) and discontinuous cell layer (Fig. 5b16). Cell membrane destruction could be also observed on PLLA/5-FU and PLLA/Bmattacin2 (Fig. 5b14). These results indicate all three kinds of composite membranes PLLA/Bmattacin2, PLLA/5-FU and PLLA/5-FU/Bmattacin2 can efficiently inhibit the growth of cancer cell HCT116.

**Figure 6:**
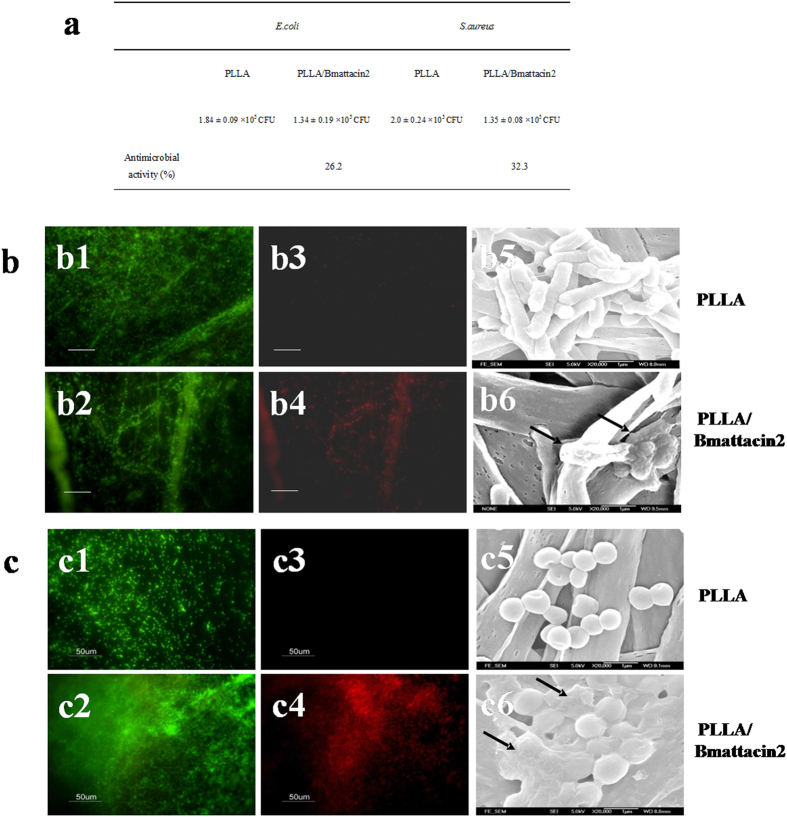
Antibacterial effect of electrospun composite membranes. (**a**) Investigating antibacterial effect of electrospun composite membranes on *E. coli* and *S. aureus* by AATCC100 method. (**b**) Observation of *E. coli* cultured on the surface of PLLA or PLLA/Bmattacin2 membranes. (b1–b4) Live/dead bacterial staining. (b5,b6) SEM observation. (**c**) Observation of *S. aureus* cultured on the surface of PLLA or PLLA/Bmattacin2 membranes. (c1–c4) Live/dead bacterial staining. (c5,c6) SEM observation.

The antimicrobial activity of electrospun PLLA/Bmattacin2 membrane on *E. coli* and *S. aureus* was determined by AATCC100. The analysis indicated PLLA/Bmattacin2 membrane had 26.2% inhibition on *E. coli* while it had 32.3% inhibitive activity on *S. aureus* (Fig. 6a). Bacterial staining by SYTO 9 and propidium iodide indicated the live (green) (Fig. 6b1) and dead bacteria (red) (Fig. 6b3) on fabricated membranes, showing the antimicrobial effect of PLLA/Bmattacin2 mat. Furthermore, SEM observed that both kinds of bacteria showed their intact shape on the surface of PLLA membrane (Fig. 6b5); however, on the PLLA/Bmattacin2 membrane (Fig. 6b6), the surface of these two kinds of bacteria were destroyed, which possibly results in the final death of these bacteria. This confirms that electrospun PLLA/Bmattacin2 has obvious activity against both these two kinds of bacteria, *E. coli* and *S. aureus*.

**Figure 7:**
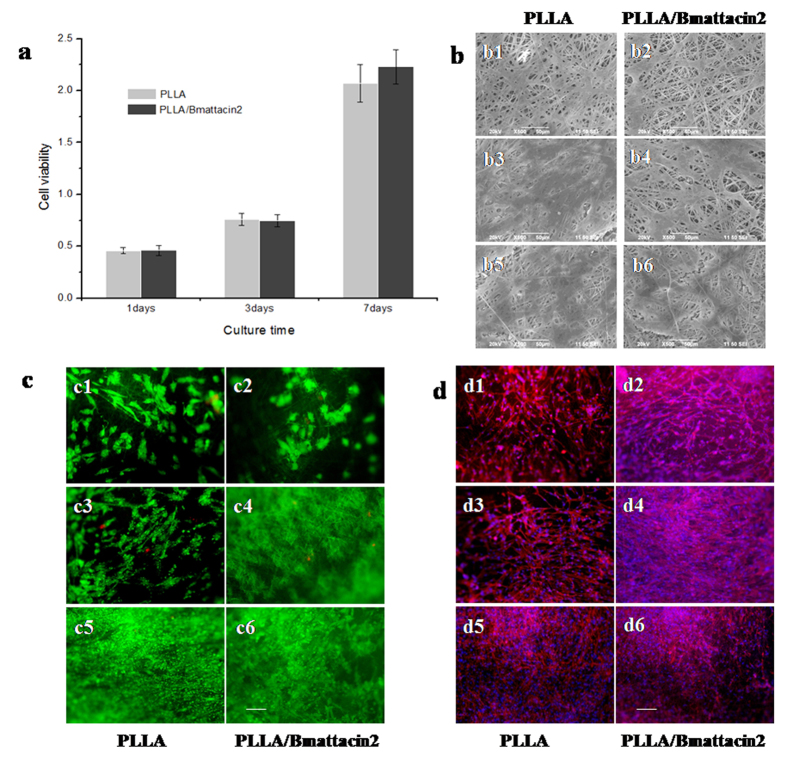
Compatibility of electrospun composite membranes. (**a**) Cell viability of HFF-1 after seeding on fabricated composite membranes for 1 day, 3 days and 7 days. (**b**) SEM observation after seeding on fabricated composite membranes for 7 days. (**c**) live/dead cell staining after seeding on fabricated composite membranes for 7 days. Bar = 50 μm. (**d**) Fluorescent staining of cells by DAPI/FITC after seeding on fabricated composite membranes for 7 days. Bar = 50 μm.

Proliferation of HFF1 cells gradually increased both on PLLA and PLLA/Bmattacin2 matrices which showed these kinds of matrices had favorable cell compatibility. In particular, at day 1, day 3, and day 7, there was no disparity of proliferation rate between these two membranes (Fig. 7a). Further, SEM was used to observe morphology of cultured HFF1 cells on PLLA and PLLA/Bmattacin2 matrices and the results implied a good compatibility of both PLLA and PLLA/Bmattacin2 membranes (Fig. 7b1). Fluorescent staining also indicated live HFF-1 cells spread all over on two kinds of matrices and scarcely any dead cells were found after several days’ cultivation (Fig. 7c,d).

PLLA/Bmattacin2 composite membrane provided a short diffusion path of protein from scaffold to encountered cells; however, in the preparation of membrane, the organic solution used in electrospinning had a negative influence on the activity of Bmattacin2. Compared with that of pure Bmattacin2, the antibacterial activity of PLLA/Bmattacin2 nanofibrous membrane decreased and the anticancer effect fall down sharply (Fig. 5a), an alternative method to electrospinning for the preparation of composite membrane must be introduced further. There has been an interesting finding that PLLA loaded Bmattacin2 together with anticancer drug 5-FU notably increase the effect of anticancer cell activity compared with that of PLLA loaded with each one of them only. This is possibly related to the mechanism of Bmattacin2 on cancer cells that with the positive charge at pH 7.0, Bmattacin2 can contribute to form channels on the membrane of cancer cells. On the one side, these channels could cause the death of cancer cells directly because of the loss of cell components. On the other side, these channels provide a way for drugs to be delivered into the cells and make the drugs more efficient. In the future, the detailed mechanism must be studied.

## Conclusions

In conclusion, a silkworm antimicrobial peptide Bmattacin2 was expressed. This recombinant peptide has anticancer cell and antibacterial properties. In order to prepare composite membranes for biomedical application, electrospinning was applied to integrate Bmattacin2 into PLLA membrane. The electrospun PLLA/Bmattacin2 membrane had good compatibility which could improve the attachment and proliferation of human skin cell HFF1. It also exhibited the expected antimicrobial effect on the bacteria, *E. coli* and *S. aureus*, which would be helpful in avoiding infection thereby accelerating skin wound healing. In addition, significant anticancer cell activity of PLLA/Bmattacin2 and the synergetic role of Bmattacin2 in composite PLLA/5-FU/Bmattacin2 membranes were observed. With these characteristics, PLLA/Bmattacin2 nanomembrane has a great potential for biomedical application in skin reconstruction, cancer therapy, wound dressing and healing.

## Methods

### The expression and purification of Bmattacin2

Full cDNA sequence and predicted amino acid sequence of *Bmattacin2* (BGIBMGA002739-TA) was downloaded from the online silkworm genome database (http://silkworm.swu.edu.cn/silkdb/). The signal peptide of Bmattacin2 was predicted on SignalP website (http://www.cbs.dtu.dk/services/SignalP/) and then was removed from the downloaded sequences. The *Bmattacin2* gene encoding mature peptide was clone into the prokaryotic expression vector pET-28a(+) (Supplementary Fig. S6) and expressed in *E. coli* BL21. SAS-PAGE was used to detect recombinant protein according to protocol. For purification, 1 liter LB was used to culture the *E. coli* cells containing recombinant plasmid, after four hours’ induction, *E. coli* cells were harvested by centrifugation at 5,000× g for 10 min. The cell pellet was washed twice, and then suspended in cold 20 mM Tris-HCl and lyzed by sonication. The lysate was centrifuged for 30 min at 14,000× g at 4 °C and the pellet was dissolved in 6 M urea. After the centrifugation at 14,000× g for 30 min at 4 °C, the supernatant was collected and filtered. His Trap affinity column (GE Healthcare) was used to purify recombinant Bmattacin2 following the instructions. Amicon Ultra-15 Centrifugal Filter (Millipore) was used for protein dialysis and concentration, finally, recombinant Bmattacin2 was stored in PBS, pH 8.0.

### Anticancer cell activity of recombinant Bmattacin2

Human foreskin fibroblasts (HFF-1, ATCC^®^ SCRC1041™) and fetal human colon cells (FHC, ATCC^®^ CRL-1831™) were purchased from ATCC. Human skin malignant melanoma cells (A375) were kindly given by Prof. Leung Yun-Chung from Department of Applied Biology&Chemical Technology of The Hong Kong Polytechnic University. Human colon cancer cell were obtained from Cell Bank of the Chinese Academy of Science (Shanghai, China). HFF-1 and A375 cells were cultured in Dulbecco’s Modified Eagle’s medium (DMEM), HCT116 cells were maintained in RPMI1640 (Roswell Park Memorial Institute 1640) while FHC were incubated in Dulbecco’s Modified Eagle Medium: Nutrient Mixture F-12 (DMEM/F12). All culture medium were supplied with 1% Penicillin Streptomycin and 10% fetal bovine serum (FBS). All culture medium and supplies mentioned above were purchased from Life technologies. DMEM/F12 were supplied with extra 10 mM of HEPES (final concentration is 25 mM), 10 ng/ml of cholera toxin, 5 μg/ml of insulin, 5 μg/ml of transferrin and 100 ng/ml of hydrocortisone. All the supplies were purchased from Sigma. The condition of cultivation was set as 37 °C, 5% CO_2_ and 95% humidity. For scanning electric microscopy observation, cells were seeded in glass disks placed in 24-well plates at the concentration of 1 × 10^4^ cells/cm^2^ and 6 × 10^4^ cells/cm^2^ and allowed to grow for 24 hours. After incubation of Bmattacin_2_ for 24 hours, cells on glass disks were fixed with 4% paraformaldehyde for 15 min at room temperature followed by dehydration with a series of graded ethanol/water solutions (50%, 70%, 80%, 95% and 100% respectively). Samples were kept in fume hood to dry at room temperature. Samples were coated with gold before observation under a scanning electron microscope (JEOL Model JSM-6490) to determine their surface morphology. For intracellular observation, cells were seeded in petri dishes until confluent was reached. They were gently washed with 37 °C phosphate buffered saline. Cells were then fixed with 4% paraformaldehyde in phosphate buffered saline at room temperature for 15 minutes, and permeabilized with 0.1% Triton X-100 in phosphate buffered saline for 5 minutes at room temperature. Then cellular skeleton were labeled by 10 μg/ml TRITC-phalloidin (Sigma) for 30 minutes, protected from light. The nucleus of cells was additionally counterstained with 1 μg/ml DAPI (Sigma) in PBS for 5 minutes. Stained cells were visualized using fluorescent microscope (Eclipse 80i, Nikon). Fluorescent images were taken by digital camera (DXM 1200C, Nikon). MTS assay (CellTiter 96^®^ AQ_ueous_ One Solution Cell Proliferation Assay, Promega) was carried out to measure the cell viability of four cell lines in response to the recombinant protein Bmattacin2. Cells were seeded on 96-well plates for around 24 hours to reach 70% confluence. After washing with PBS, cells were incubated with different concentration of Bmattacin2 (1–12 μM) respectively for 24 hours. Medium was removed and the cells were washed with fresh PBS. 100 μl of fresh medium was added to each well. MTS solution was added to each well in the ratio of 1:5. The plates were incubated at 37 °C/5% CO_2_ for 3–4 hours followed by the recording of the OD values of the medium evaluated at 492 nm using Micro-plate Reader (Infinite F200, TECAN). Cell viability was expressed as mean ± SD, calculated from three independent experiments.

### Antimicrobial activity of recombinant Bmatacin2

The minimum inhibitory concentrations (MIC) of Bmattacin2 on five bacteria strains were measured using a broth micro-dilution assay with a minor modification^38^,^39^,^40^,^41^. Five bacteria strains were used including *Escherichia coli* ATCC25922, *Staphylococcus aureus* ATCC25923, *Seiiatiomaicesceis, Escherichia coli* DH5α and *Bacillus bombysepticus.* Single colony of each bacteria strain was inoculated in 5 ml LB at 37 °C overnight, then 50 μl of culture was added to 5 ml LB for 3–4 hours’ incubation until the OD reached 0.4, the bacterial concentration then was diluted to 1.5 × 10^8^ colony-forming unit(CFU)/ml according to Mcfarland standard 0.5. Serial dilutions of purified Bmattacin2 were transferred to 96-well plate together with bacteria culture to ensure the final concentration of bacteria was 5 × 10^4^ CFU/well. The whole volume of Bmattacin2 and bacteria mixture was 100 μl. The MIC values were defined as the lowest concentration of Bmattacin2 which maintained the culture medium clear after 18–24 hours of incubation. Referring to the protocol from Cado’s study^42^, after minor modification, the normalized growth rate of bacteria was plotted by OD_600_ value in the presence or absence of Bmattacin2. Data were expressed as mean with standard deviation of at least three replicates. The OD_600_ value of cultures in the absence of Bmattacin2 (negative control) was taken as 100% bacteria growth and the OD_600_ value of culture medium was taken as 0% growth (blank). The corresponding bacterial growth rates were evaluated and calculated by the following equation:


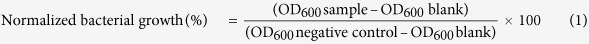


The Live/dead status of bacteria under a fluorescence microscope was observed. The concentration of bacteria was adjusted to about 2 × 10^8^/ml according to the Mcfarland standard. Cells were centrifuged at 10,000 rpm for 5 minutes and the pellet was suspended in HEPES (4-(2-Hydroxyethyl)-1-piperazineethanesulfonic acid, 10 mM) buffer. The bacterial suspension (1 ml) was mixed with 3 μl of pre-mixed dye of SYTO9 and propidium iodide (Live/dead BacLight Bacterial Viability assay, Life technologies), and incubated at room temperature in the dark for 15 minutes. For the surface morphology of bacteria, Bmattacin2 in the concentration of 10 μM was applied to a colony of *E. coli* ATCC25922 or *S. aureus* ATCC25923 in aluminum foil. Afterwards, the mixture was air-dried and coated with gold. The coated mixture was subjected to scanning electron microscopy.

### Measurement of zeta potential of cells and binding affinity of Bmattacin2

For zeta potential measurement, the concentration of bacteria (*E. coli* ATCC25922 or *S. aureus* ATCC25923) was adjusted to about 1.5 × 10^8^/ml according to the Mcfarland standard 0.5. The zeta potentials of four human cells were measured as well, 1 × 10^6^ cells were incubated in the presence of Bmattacin2 for 30 minutes with different concentrations. After washing with HEPES, the bacteria or human cells were re-suspended again and treated with Bmattacin2 (dissolved in HEPES as well) in different concentrations. The pH value was monitored and maintained at 7.4. The suspension was dispersed into disposable zeta cells with gold electrodes and allowed to equilibrate for 30 min at 25 °C. The zeta potential was measured using a zeta potential analyzer (Zeta Plus) from Brookhaven Instruments Corporation. The value was automatically calculated from electrophoretic mobility based on the Smoluchowski equation^43^,^44^. The binding affinity of Bmattacin2 was determined by visualizing cells in the presence of FITC labeled Bmattacin2. Cells were seeded on cell chambers until 70% confluent, 0.5 μM FITC-Bmattacin2 was then added for 24 hours, after thoroughly wash, cytoskeleton were stained by TRITC-phalloidin. Cells and remaining FITC-Bmattacin2 were observed under fluorescence microscopy.

### Preparation of composite membranes by electrospinning

A PLLA solution with a concentration of 1.0% was prepared by dissolving PLLA in a mixed organic solution of chloroform (90 wt%) together with DMF (10 wt%). 1 mg prepared Bmattacin2 powder was dispersed in 5 g PLLA solution to form the PLLA/Bmattacin2 suspension. A syringe with a needle which was connected to high-voltage electricity was used to load and deliver this suspension. Electrospinning was carried out at a voltage of 15 KV, a flow rate of 0.3 ml/min and a 10 cm distance from the needle tip to the receptor, a grounded aluminum foil. Meanwhile, pure PLLA nanofibrous membrane was also prepared as a control based on the same parameters. For observation of microstructure of electrospun membranes, samples were compressed to films together with potassium bromide power respectively, and then their microstructures were investigated by Fourier transform infrared spectrometry (FTIR, Nicolet 5700, Thermo Co.). All spectra were recorded in absorption mode at 2 cm^−1^ interval wavenumbers from 500 cm^−1^ to 2500 cm^−1^. The morphology of electrospun nanofibers was observed by a scanning electron microscope (SEM) (Stereoscan 440, LEICA). The diameter of electrospun nanofibers were measured by Nano Measurer 1.2 by selecting fibers randomly at more than 3 different locations. Totally more than 100 different fibers were measured for each sample. All collected data were analyzed by OrginPro8 (OriginLab Corporation). The values were reported as the mean ± SD for all of the results. For measurement of mechanical property, the electrospun membrane were cut into 50 × 10 mm strips respectively and then mechanical performance of these membranes were characterized by Instrons 5560 (Instron, Canton, MA) with a load cell of 10 N. Each tensile test was performed at room temperature with a crosshead speed of 5 mm/min and a preload of 0.1 MPa. All reported tensile strength and tensile stress values represent the average of 7–8 measurements. All collected data were analyzed by OrginPro8 (OriginLab Corporation). The values were reported as mean ± SD for all of the results.

#### Characterization of PLLA/Bmattacin2 membranes

For investigation of anticancer-cell activity, the lyophilized Bmattacin2 and 5-FU (sigma) was blended into PLLA solution and the electrospun PLLA/Bmattacin2 (2 wt%), PLLA/5-FU (7.5 wt%), and PLLA/5-FU/Bmattacin2 (7.5 wt%, 2 wt%) membrane were prepared. Fibrous membranes were placed in multi-well plates. After UV exposure, the cells were seeded at the density of 6 × 10^4^ cells per cm^2^. Cells in the wells were washed with PBS before evaluation. The MTS solution and 100 μl of fresh medium were added to each well at the ratio of 1:5, the plates were incubated at 37 °C for 4 hours. The absorbance of medium at 490 nm was recorded by micro-plate reader. Data are mean with standard deviation from triplicate determinations. ANOVA was applied for multi-group comparison followed by the Least Significant Difference (LSD) test for means comparison between the groups. For live/dead staining, cell permeable esterase-substrate fluorescein diacetate (FDA) together with cell nucleic acid stain propidium iodide (PI) were used to assess the viability of cells grown on the nanofibrous membrane. Each sample was washed by PBS for three times and then the cells were stained by rinsing in 1 ug/ml FDA and 1 ug/ml PI for 5 minutes at dark and room temperature. Then samples were observed by fluorescent microscope. The assessment of intracellular structure staining and surface morphology observation of PLLA/Bmattacin2 membranes were conducted using the same methods mentioned at the session of anticancer cell activity of recombinant Bmattacin2. AATCC100 was used to determine activity of electrospun PLLA/Bmattacin2 membrane. The electrospun PLLA/attacin2 membrane (Concentration of Bmattacin2 is 2 wt%) and PLLA membrane (negative control) were cut into pieces with size of 2.5 cm^2^. After UV sterilization, two pieces were placed into each well of 6-well tissue culture plate. Single colony of each bacteria strain (*E. coli* and *S. aureus*) was inoculated in 5 ml LB at 37 °C overnight, then 50 μl of culture was added to 5 ml LB for 3–4 hours’ incubation until the OD_600_value reached 0.4, then the bacterial concentration was diluted to 1 × 10^5^–2 × 10^5^ CFU/ml. 500 μl bacterial culture was added to each well and then the plate was incubated at 37 °C for 4 h. After incubation, culture in each well was neutralized with 4 ml iced saline (0.85%). 50 μl of mixture of each well was spread on LB agar plate and incubate at 37 °C overnight. The colonies on each agar plate were counted and the antimicrobial effect of each sample was calculated by the following formula:


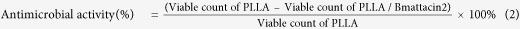


Each kind of sample had another two repetitions. All collected data were analyzed by OrginPro8 (OriginLab Corporation). The values were reported as mean ± SD for all of the results. For live/dead bacterial staining, the electrospun PLLA/attacin2 membrane and PLLA membrane (Negative control) coated on glass disks (1 cm^2^) were prepared. After UV sterilization, glass disks with membrane were placed into wells of 24-well tissue culture plate. Single colony of each bacteria strain (*E. coli and S. aureus*) was inoculated in 5 ml LB at 37 °C overnight, then 50 μl of culture was added to 5 ml LB for 3–4 hours’ incubation until the OD_600_ value reached 0.4 and then bacterial concentration was diluted to 1.5 × 10^8^ CFU/ml according to the Mcfarland standard 0.5. 100 ul bacterial culture was added on the center of each glass disk and then the plate was cultured at 37 °C for 4 h. The bacteria on membrane were stained by SYTO 9 and propidium iodide and then were observed by fluorescent microscope. The morphology of bacteria on membranes was observed by SEM. For investigation of compatibility, the electrospun PLLA/Bmattacin2 membrane and PLLA membrane coated on glass disks (1 cm^2^) were prepared. After UV sterilization, glass disks with membrane were placed into wells of 24-well tissue culture plate. HFF-1 cells were seeded on PLLA and PLLA/Bmattacin2 membrane in 24-well plate at the concentration of 6 × 10^4^ cells/cm^2^ and then were incubated at 37 °C for 4 hours to allow cell attachment. 1 ml growth medium was added to each well and then incubated for total 1d, 3d and 7d. After incubation, MTS assay, live/dead staining, intracellular staining and surface morphology of cells on the membrane were evaluated by the same methods mentioned at anti-cancer cell activity evaluation.

## Additional Information

**How to cite this article**: Li, Z. *et al.* Composite Membranes of Recombinant Silkworm Antimicrobial Peptide and Poly (L-lactic Acid) (PLLA) for biomedical application. *Sci. Rep.* **6**, 31149; doi: 10.1038/srep31149 (2016).

## Supplementary Material

Supplementary Information
