# Supplementary material for: Composite Membranes of Recombinant Silkworm Antimicrobial Peptide and Poly (L-lactic Acid) (PLLA) for biomedical application
Source: Sci Rep. 2016 Aug 9;6:31149. doi: 10.1038/srep31149 (PMC4977571; doi:10.1038/srep31149)
Supplement: Supplementary Information [file srep31149-s1.doc]

Supplementary Information

**Composite Membranes of Recombinant Silkworm Antimicrobial Peptide and Poly (L-lactic Acid) (PLLA) for biomedical application**

*Zhi Li, Xuan Liu, Yi Li^*^, Xiqian Lan, Polly Hangmei Leung, Jiashen Li, Gang Li, Maobin Xie, Yanxia Han, Xiaofen Lin*


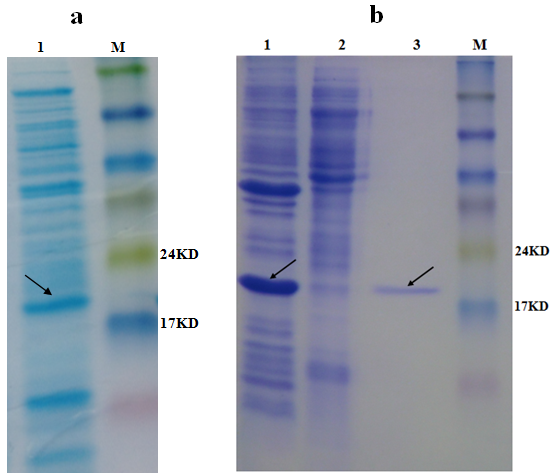


**Figure S1.** Expression (a) and purification of *Bmattacin2*. (a) Lane 1, Expression of *Bmattacin2* in *E.coli*. Lane M, Protein maker. (b) Lane 1, Pellet after treated with urea solution. Lane 2, Washed pool. Lane 3, Eluted pool. Lane M, Protein marker. Arrows indicate the target protein bands.


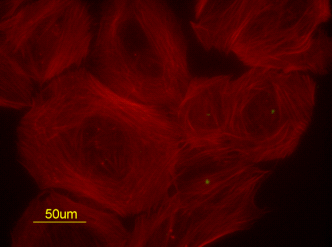

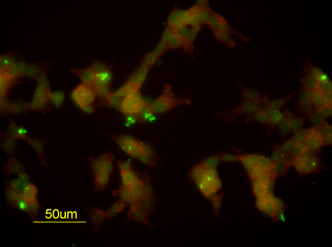

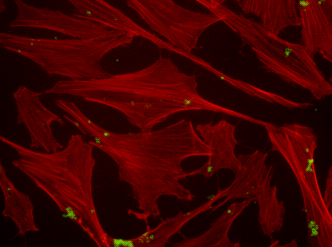

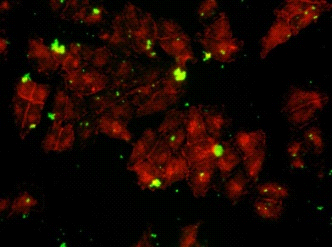


**FHC**

**HFF**

**-**

**1**

**A375**

**HCT116**

**Figure S2.** The binding affinity of Bmattacin2 was determined by visualizing cells in the presence of FITC labeled Bmattacin2. Four cell lines were observed. Cells were seeded on cell chambers until 70% confluent, 0.5 μM FITC-Bmattacin2 was then added for 24 hours, after thoroughly wash, cytoskeleton were stained by TRITC-phalloidin. Cells and remaining FITC-Bmattacin2 were observed under fluorescence microscopy. Bar = 50 μm.


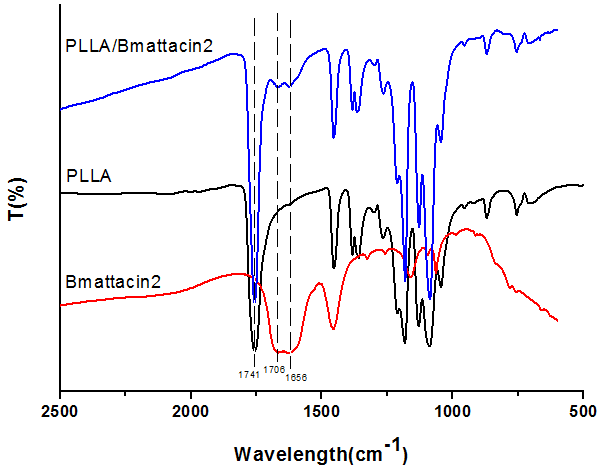


**Figure S3.** FTIR spectra of PLLA/Bmattacin2 membrane, PLLA membrane and Bmattacin2 powder.

**a**

**b**

**Figure S4.** Diameter distribution of PLLA (a) and PLLA /Bmattacin2 fibers (b).


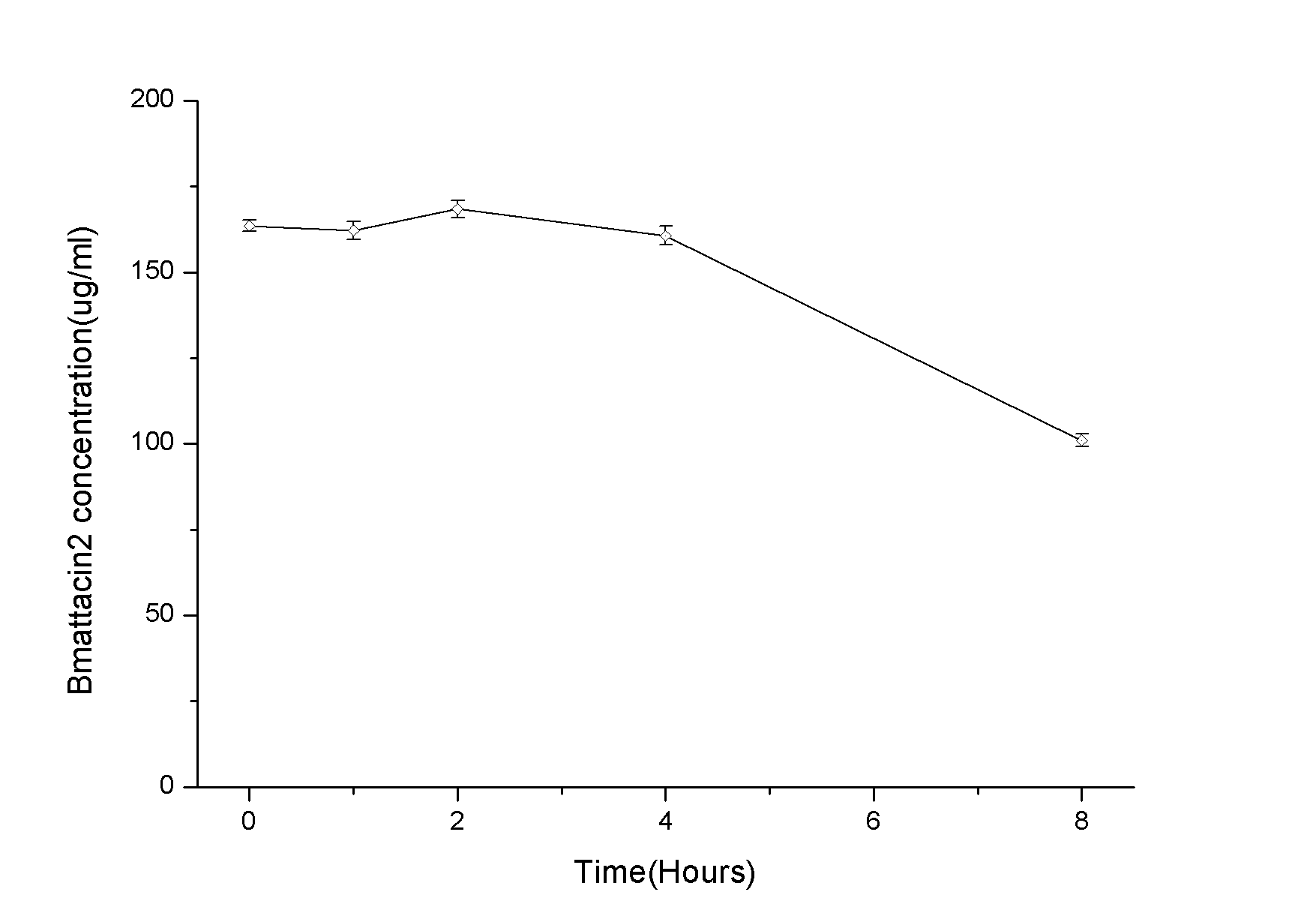
**
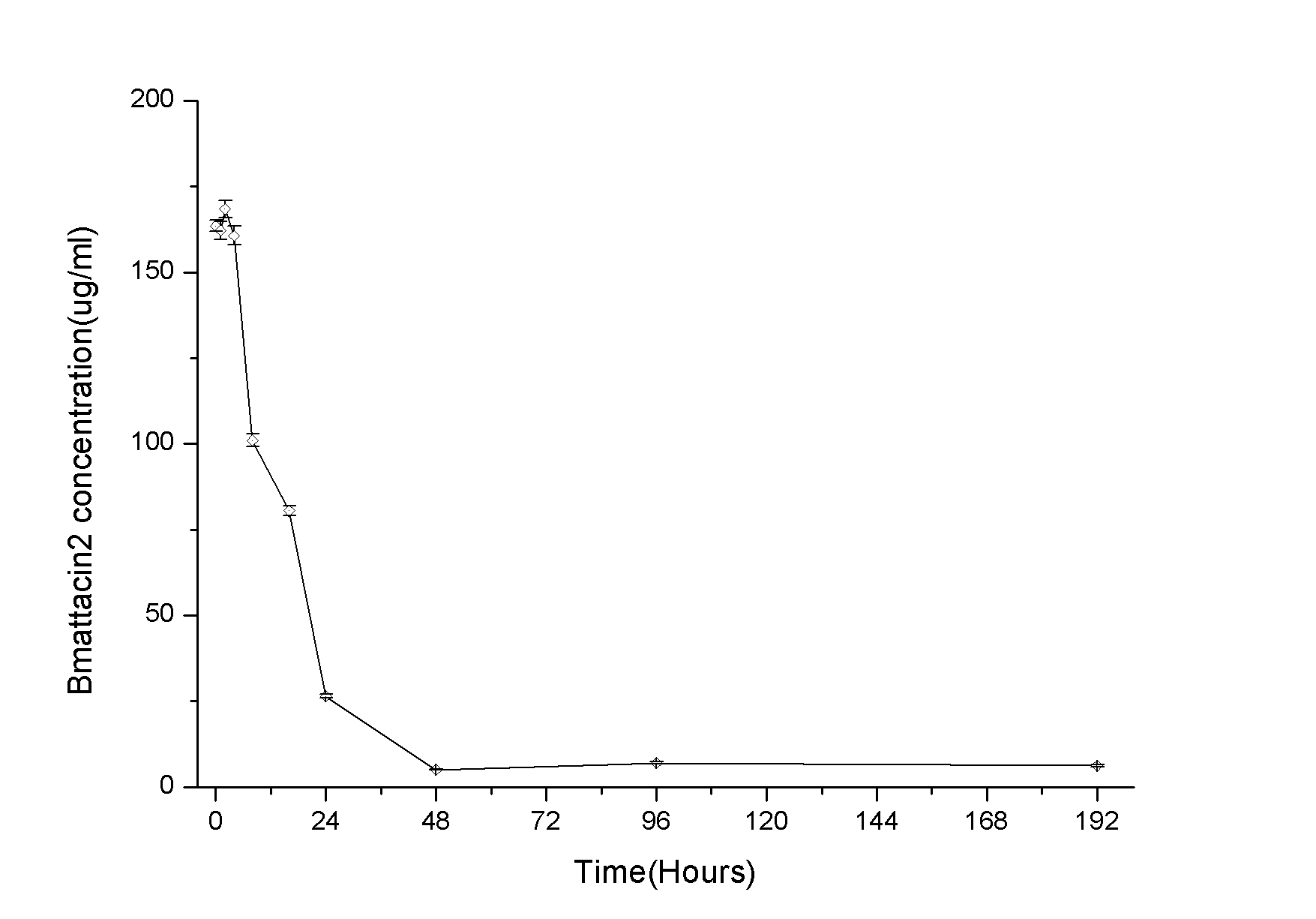
 Figure S5.** Release property and degradation of Bmattacin2 in electrospun membrane.

**Figure S6.** Schematic representation of expression vector construction for heterologous expression

**Table S1** Tensile strength of PLLA and PLLA/Bmattacin2 membrane

| Membrane | Maximum load  (cN) | Tensile strength  (MPa) | Tensile  strain  (%) | Young's modulus  (MPa) |
| --- | --- | --- | --- | --- |
| PLLA | 35.9±6.2 | 3.6±0.6 | 11.8±2.7 | 123.8±21.0 |
| PLLA/Bmattacin2 | 27.1±2.8 | 2.7±0.3 | 24.2±3.9 | 45.7±13.2 |
